# Temperature Dependence of Raman-Active In-Plane E_2g_ Phonons in Layered Graphene and h-BN Flakes

**DOI:** 10.1186/s11671-018-2444-2

**Published:** 2018-01-17

**Authors:** Xiaoli Li, Jian Liu, Kai Ding, Xiaohui Zhao, Shuai Li, Wenguang Zhou, Baolai Liang

**Affiliations:** 1 College of Physics Science & Technology, Hebei University, Baoding, 071002 People’s Republic of China; 2 State Key Laboratory of Superlattices and Microstructures, Institute of Semiconductors, Chinese Academy of Sciences, Beijing, 100083 People’s Republic of China

**Keywords:** Graphenes, h-BNs, In-plane E_2g_ phonon, Temperature dependence

## Abstract

Thermal properties of sp^2^ systems such as graphene and hexagonal boron nitride (h-BN) have attracted significant attention because of both systems being excellent thermal conductors. This research reports micro-Raman measurements on the in-plane E_2g_ optical phonon peaks (~ 1580 cm^−1^ in graphene layers and ~ 1362 cm^−1^ in h-BN layers) as a function of temperature from − 194 to 200 °C. The h-BN flakes show higher sensitivity to temperature-dependent frequency shifts and broadenings than graphene flakes. Moreover, the thermal effect in the c direction on phonon frequency in h-BN layers is more sensitive than that in graphene layers but on phonon broadening in h-BN layers is similar as that in graphene layers. These results are very useful to understand the thermal properties and related physical mechanisms in h-BN and graphene flakes for applications of thermal devices.

## Background

Both graphene and hexagonal boron nitride (h-BN) flakes have a layered structure, with weak Van der Waals (vdW) interactions keeping the layers together but strong sp^2^ chemical bonds making atoms held together within each layer [1, 2]. Due to the layered structure, these two materials are excellent thermal conductors [3, 4] and their thermal properties have attracted significant attention [5, 6]. The thermal transports in them are dominated by lattice vibrations and are described properly by phonon scattering [7–9]. There is a Raman-active mode with symmetry E_2g_ describing in-plane atoms movement, which is named as G peak [10, 11] in graphene layers and E_2g_^high^ peak [12, 13] in h-BN layers (distinguishes from the low frequency E_2g_ mode at about 53 cm^−1^ [14, 15], denoted as E_2g_^low^). The frequency shifts and broadenings of these two-phonon scattering peaks are dependent on the elongation of the intra-layer C–C bond (or B–N bond) and meanwhile the number of layers [16, 17] due to thermal expansion or multi-phonon anharmonic couplings [9, 18, 19]. Thus, the in-plane E_2g_ phonon plays an important role in the study of thermal properties of sp^2^ materials. Several papers have reported the temperature dependence of the frequency or linewidth of G peak or E_2g_^high^ peak in the Raman spectra of ultrathin graphene layers [9, 16, 17], bulk graphite [9, 18], and bulk h-BN [14, 19], respectively. However, the temperature effect on in-plane E_2g_ phonon in graphene as well as h-BN layers and the thermal properties of these two materials still lack of a detailed comparison.

In this research, we measured G peak in graphene layers and E_2g_^high^ peak in h-BN layers by micro-Raman spectroscopy at the temperature range from − 194 to 200 °C. Temperature dependence of frequency shifts and broadenings of these two peaks were investigated in graphene and h-BN layers with similar thickness. Furthermore, the thermal effect in the c direction on their frequency shifts and broadenings was studied in graphene and h-BN layers as thickness increases. A similar comparison has not yet been reported previously. Therefore, Raman microscopy is a very useful tool to investigate the thermal properties for micro-scale flakes of graphene- and h-BN-layered structures.

## Experimental

**Fig. 1:**
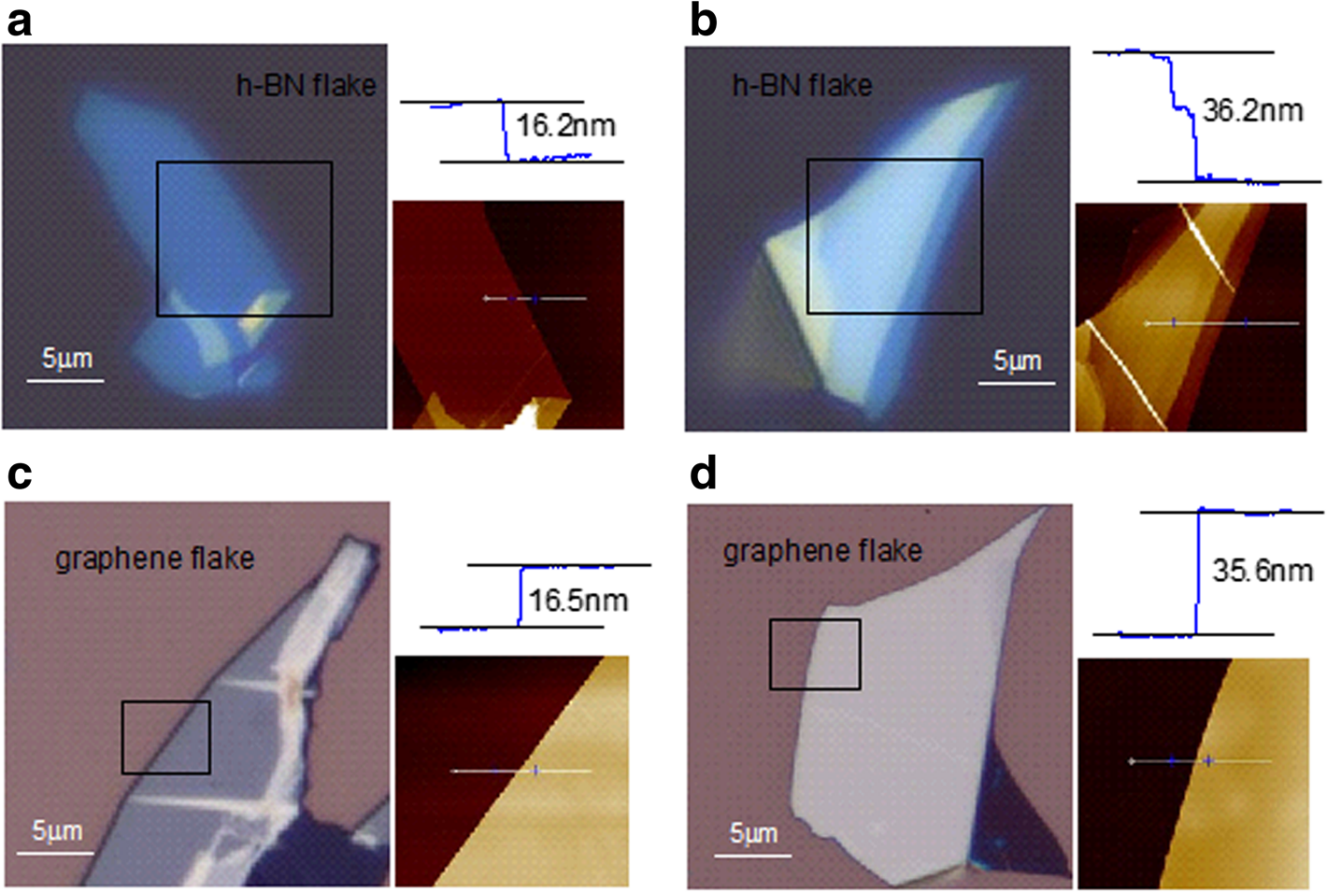
**a**–**d** Optical images of the selected h-BN and graphene flakes on the SiO_2_/Si substrate. Additional insets give the respective AFM image and sample thickness of the highlighted *black rectangle* areas in optical images

Graphene flakes and h-BN flakes were obtained by micromechanical cleavage of bulk graphite crystals and bulk single-crystalline BN platelet on SiO_2_/Si substrate with SiO_2_ thickness as 90 nm. Layered graphenes and h-BNs can be easily seen under a microscope. We selected some flakes with dozens of atomic layers to avoid the higher influence of adsorbates and charge transfer from SiO_2_/Si substrate [8] and to eliminate the heating enhancement in ultrathin graphene and h-BN layers. The thickness of graphene flakes and h-BN flakes was determined by atomic force microscopy (AFM) measurement with a tapping mode. Figure 1 shows the microscope images of four selected h-BN and graphene flakes, and their AFM images as well as the thickness measured in the black rectangles highlighted in microscope images. Figure 1a, b shows two h-BN flakes with the thickness of 16.2 and 36.2 nm, and Fig. 1c, d shows two graphene flakes with the thickness of 16.5 and 35.6 nm, respectively. They are selected to have similar thickness in order to facilitate the comparison for temperature dependence of frequency shifts and broadenings of phonons in micro-Raman spectroscopy.

The temperature-dependent Raman spectra of G peak and E_2g_^high^ peak were measured in back-scattering with a HR Evolution micro-Raman system, equipped with the unique SWIFT™ CCD, a × 50 objective lens (NA = 0.45). The samples were mounted on an in-house-made sample holder consisting of a thin copper disk with a central pillar and a 500-μm diameter hole. Measurements from − 194 °C to 200 °C were carried out in a liquid nitrogen (LN_2_) cooled low-temperature Linkam stage equipped with a temperature controller. All spectra were excited with a 532-nm laser and recorded with an 1800 lines/mm grating to enable each pixel of the charge-coupled detector to cover 0.5 cm^−1^. A laser power below 2 mW was used to avoid sample heating. The integration time of 20 s was adopted to ensure a good signal-to-noise ratio.

## Results and Discussion

**Fig. 2:**
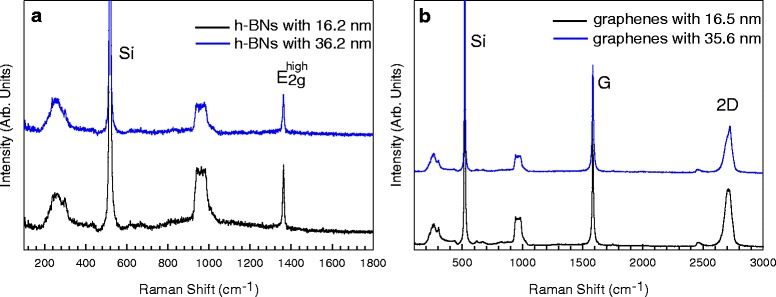
**a**, **b** Raman spectra of h-BN and graphene flakes at room temperature. The *blue curves* are vertically shifted for clarity

The G peak and E_2g_^high^ peak are representative in-plane Raman modes. We first illustrated Raman spectra of the four selected flakes (shown in Fig. 1) at room temperature in Fig. 2, in which the curves from bottom to top are given in the order of increasing thickness and the curves are offset for clarity. Figure 2a shows Raman spectra of h-BN flakes in the spectral range from 100 to 1800 cm^−1^. The peaks at about 300, 520, and 940 cm^−1^ are characteristic peaks of Si substrate [20], and the E_2g_^high^ peak is at about 1362 cm^−1^. The frequency of the E_2g_^high^ peak is almost the same in two flakes. Yet the Si peaks at the 36.2 nm h-BN flake are weaker than that at the 16.2 nm h-BN flake due to more absorption of Raman signals in a thicker flake [21]. Figure 2b shows Raman spectra of graphene flakes in the spectral range from 100 to 3000 cm^−1^, which consists of the Si peaks of Si substrate, G and 2D peaks of graphene flakes. The positions of Si peaks are the same with those in Fig. 2a. G peak appears around 1580 cm^−1^, and 2D peak is at around 2700 cm^−1^ which is a second-order Raman mode and is another fingerprint of graphene layers [11]. The G peak exhibits no significant difference in frequency, while the intensities of Si peaks decrease as the thickness of graphenes increases. G peaks are much stronger than E_2g_^high^ peaks because resonant excitation is easy to be satisfied in graphene layers due to its zero gap [22]. The second-order Raman peaks of h-BN layers have not been obtained for the reason that Raman processes are non-resonant in h-BN layers when laser source is in the visible range [23]. There are no defective Raman peaks in h-BN and graphene layers, meaning that these flakes are defect-free crystals, which are suitable prototype systems to study the temperature dependence of in-plane E_2g_ phonons.

**Fig. 3:**
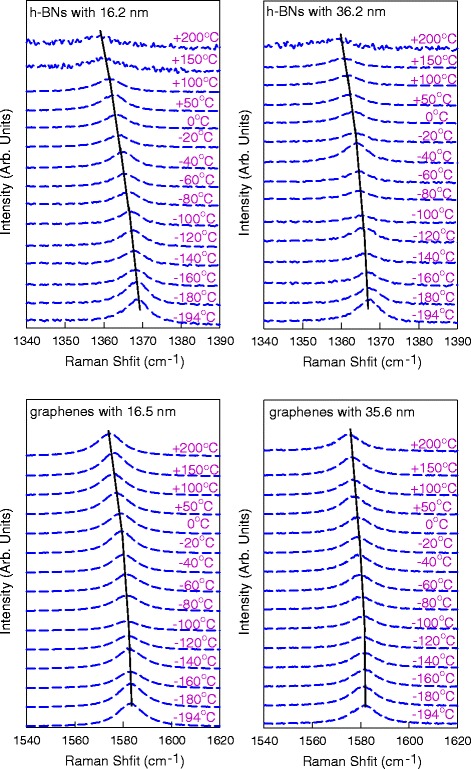
Intensity-normalized Raman spectra of E_2g_^high^ peaks in h-BN flakes and G peaks in graphene flakes for the temperature range of − 194 ~ 200 °C. The curves are vertically shifted for clarity

We further measured variable temperature Raman spectra of G peak or E_2g_^high^ peak on chosen four flakes in the temperature range of − 194~200 °C, as shown in Fig. 3. It is obvious that both G peaks and E_2g_^high^ peaks show a progressive downshift as the temperature increases. The Raman peaks were fitted by a single Lorentzian profile to obtain their frequencies and full width at half maximum (FWHMs).

**Fig. 4:**
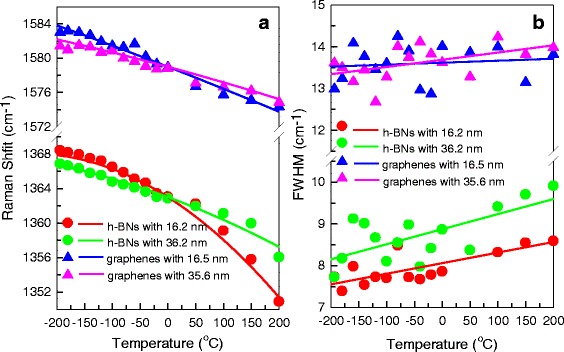
**a**, **b** The Raman shift and FWHM of E_2g_^high^ peaks in h-BN flakes and G peaks in graphene flakes for the temperature range of − 194 ~ 200 °C

Figure 4a shows the frequency shifts of G peak and E_2g_^high^ peak. In theory, the temperature dependence of phonon pulsation ω_ph_ in both E_2g_^high^ peak and G peak indicates a nonlinear relationship, which can be described by fitting a second-order polynomial, ω_ph_ = ω_ph_^0^ + at+bt^2^ [18, 19]. Here, ω_ph_^0^ is the phonon frequency at 0 °C. The thermal frequency shifts are best fitted, and the constants of ω_ph_^0^, a, and b are given in Table 1. We obtained some results from these constants.

**Table 1:** Temperature dependence of Raman frequency of E_2g_^high^ peak and G peak are fitted by ω_ph_ = ω_ph_^0^ + at+bt^2^, and the constants of ω_ph_^0^, a, and b are given in the table

Flake	Phonon	Thickness	ω_ph_^0^	a	b
h-BNs	E_2g_^high^	16.2 nm	1363 cm^−1^	− 0.04123 cm^−1^ °C	− 8.446 × 10^−5^ cm^−1^ °C^2^
	E_2g_^high^	36.2 nm	1363 cm^−1^	− 0.02385 cm^−1^ °C	− 2.501 × 10^−5^ cm^−1^ °C^2^
Graphenes	G	16.5 nm	1579 cm^−1^	− 0.02519 cm^−1^ °C	− 5.187 × 10^−6^ cm^−1^ °C^2^
	G	35.6 nm	1579 cm^−1^	− 0.01745 cm^−1^ °C	− 7.145 × 10^−6^ cm^−1^ °C^2^

First, ω_ph_^0^ in two h-BN flakes is the same as 1363 cm^−1^ and in two graphene flakes is the same as 1579 cm^− 1^. It means that frequencies of both two E_2g_ modes are independent on thickness at about 0 °C. Their frequency differences at 25 °C are below 0.5 cm^− 1^ with different thickness, which is below the resolution of the Raman system. This is why the E_2g_^high^ peak and G peak positions show no shifting in different thickness at room temperature in Fig. 2. Second, with increasing temperature, E_2g_^high^ and G modes display a marked frequency downshift. The shifts of the E_2g_^high^ peak are − 18 and − 12 cm^− 1^ in 16.2 and 36.2 nm h-BN flakes respectively in the temperature from − 194 to 200 °C, whereas the shifts of G peak in two graphene flakes are smaller and remain below − 10 cm^−1^. This shows that the frequency shift of E_2g_^high^ peak is about 1.4–2.1 times than that of G peak in the similar thickness of h-BN and graphene flakes as the temperature varied by Δ*t* ~ 400 °C. Our experimental results can find some supporting evidence from previous calculation results. In the reference [18] and [19], frequency shifts of E_2g_ phonon are calculated in bulk h-BN [19] and bulk graphite [18] by three-phonon, four-phonon, and thermal expansion contributions. The frequency shift of E_2g_^high^ peak in bulk h-BN from 100 to 600 K is about − 10 cm^−1^ [19], but that of G peak in bulk graphite from 100 to 600 K is about − 5 cm^−1^ [18]. We can see that multi-phonon coupling plays a major role on frequency shifts. Thus, h-BN flakes show higher sensitivity to temperature-dependent frequency shifts than graphene flakes, which should be attributed to stronger multi-phonon coupling in h-BN flakes.

Figure 4b shows the FWHMs of G peak and E_2g_^high^ peak. In the temperature range of interest here, the linewidth of both modes indicates a linear relationship. Similar behavior has been reported for bulk h-BN with temperature below 400 K [19]. We fitted the relation between temperature and FWHM by a first-order polynomial, *Γ*_ph_ = *Γ*_ph_^0^ + ct, where *Γ*_ph_^0^ is the FWHM at 0 °C. The constants of *Γ*_ph_^0^ and c are given in Table 2. Some results can be seen from these constants.

**Table 2:** Temperature dependence of FWHM of E_2g_^high^ peak and G peak are fitted by *Γ*_ph_ = *Γ*_ph_^0^ + ct, and the constants of *Γ*_ph_^0^ and c are given in the table

Flake	Phonon	Thickness	*Γ*_ph_^0^	c
h-BNs	E_2g_^high^	16.2 nm	13.62 cm^− 1^	4.707 × 10^−4^ cm^−1^ °C
	E_2g_^high^	36.2 nm	13.69 cm^− 1^	1.727 × 10^−3^ cm^−1^ °C
Graphenes	G	16.5 nm	8.061 cm^− 1^	2.533 × 10^− 3^ cm^−1^ °C
	G	35.6 nm	8.874 cm^−1^	3.604 × 10^− 3^ cm^−1^ °C

The FWHM of the E_2g_^high^ peak is 7 ~ 10 cm^−1^ in two h-BN flakes, whereas the FWHM of G peak in two graphene flakes is larger and remains 13 ~ 14 cm^−1^. They are in good agreement with the experimental results reported in bulk graphite [18] and bulk h-BN [19]. The E_2g_^high^ modes exhibit a sizable broadening of ~ 1 cm^−1^ as temperature increases; in contrast, G modes show an insignificant broadening in the temperature range studied. It means that the lifetime of the E_2g_^high^ peak is more sensitive to the temperature variation than that of G peak in the similar thickness of h-BN and graphene flakes as the temperature varied by Δ*t* ~ 400 °C. Our experimental results can be explained in terms of the calculation of the references [18] and [19]. The FWHM broadenings of E_2g_ phonon are calculated in bulk h-BN [19] and bulk graphite [18] by three-phonon and four-phonon contributions. The FWHM broadening of the E_2g_^high^ peak in bulk h-BN from 100 to 300 K is about 1.5 cm^−1^ [19], but that of G peak in bulk graphite from 100 to 300 K is about zero [18]. The multi-phonon coupling also plays a major role on FWHM broadenings. Thus, h-BN flakes show higher sensitivity to temperature-dependent FWHM broadenings than graphene flakes, which we think should be also attributed to stronger multi-phonon coupling in h-BN flakes.

In addition, with increasing thickness, the frequency shifts of both G peak and E_2g_^high^ peak become smaller. It is in good agreement with the experimental results reported in references [16, 17], in which Calizo el al. found that the shift of G peak in bilayer graphenes is larger than those in graphite as the temperature varied from 100 to 400 K [16], and the shift of G peak in monolayer graphenes is larger than that in bilayer graphenes as the temperature varied from − 200 to 100 °C [17]. In this paper, the frequency shifts related to the c direction thickness are evaluated to be − 8.9 × 10^−4^ cm^−1^/(°C nm) in h-BN layers and − 3.5 × 10^−4^ cm^−1^/(°C nm) in graphene layers, respectively, in the temperature range from − 194 to 200 °C. The frequency shift in the c direction of the E_2g_^high^ peak is ~ 2.5 times that of G peak as the temperature varied by Δ*t*~400 °C. Meanwhile, the slopes of the FWHM of both G peak and E_2g_^high^ peak have a slight increase with increasing thickness. The FWHM broadenings related to the c direction thickness are evaluated to be 5.5 × 10^−5^ cm^− 1^/(°C nm) in h-BN layers and 5.9 × 10^−5^ cm^−1^/(°C nm) in graphene layers, respectively, in the temperature range from − 194 to 200 °C. The FWHM broadening in the c direction of the E_2g_^high^ peak has the same sensitivity to temperature as that of G peak. This means that the thermal effect in the c direction on phonon frequency in h-BN layers is more sensitive than that in graphene layers but on phonon broadening in h-BN layers is similar as that in graphene layers. However, we can hardly find relevant theoretical calculations about the frequency shift and the FWHM broadening of E_2g_ phonons with increasing h-BN or graphene thickness to explain the physical mechanism of our experiment. We think our results are attributed to joint contributions of anharmonic interaction and other more complex couplings. The mechanisms are still not well understood and need further study.

## Conclusions

Graphene and h-BN layers are isoelectronic materials. Their in-plane sp^2^ structures exhibit a similar hexagonal structure with similar lattice parameters, and they are usually stacked to form a multilayer in a stable configuration of AB stacking when prepared by mechanical exfoliation. Given the similarities of an atom structure, the properties of these two materials are expected to be similar to facilitate the comparison. Raman spectroscopy is a powerful characterization tool for graphene and h-BN materials with respect to thermometry. We performed a Raman scattering study of in-plane E_2g_ phonons in layered h-BN and graphene flakes in the temperature range from − 194 to 200 °C. The frequency shifts and FWHM broadenings of E_2g_^high^ peak and G peak indicate that h-BN flakes are more sensitive to temperature than graphene flakes with similar thickness. The effect of the thermal conduction in the c direction on phonon frequency in h-BN layers is better than that in graphene layers but on phonon broadening in h-BN layers is similar as that in graphene layers. These results are very useful to further understand the thermal properties and related physical mechanisms in h-BN and graphene flakes for applications of thermal devices.

## Abbreviations

AFM: Atomic force microscopy
FWHMs: Full width at half maximum
h-BN: Hexagonal boron nitride
vdW: Van der Waals
